# Progress in Surgery

**Published:** 1896-09-12

**Authors:** 


### Progress in Surgery.

### INFECTIOUS DISEASES.

(*Continued from page* 358.)

**Malaria.**—The diseases caused by plasmodia are hardly less fatal than those due to bacilli, but our knowledge of the life-history of these parasites is at present scanty. Bruce[1] has recently discovered the cause of tsetse-fly disease, which destroys the cattle and horses over such huge areas of Eastern Africa, and causes such a demand for slaves in their stead to carry goods. The fly seems to be merely a passive agent, conveying a protozoon akin to that which produces the surra horse disease in India. This organism multiplies in the blood, produces a high degree of anæmia, and quickly kills the animal. It appears only in certain districts, has little or no effect on man, and as to what its natural habitat or host is we have no knowledge. Colonel Lawrie has[2] brought forward evidence to show that Laveran's malaria microbes are nothing but products of the degenerate blood cells which have reverted to a reptilian type under the influence of an endemic disease of the spleen. He argues that the corpuscles have never been isolated in a pure culture according to Koch's canons, and remarks that they are absent in many of the worst cases of malaria. The "swarming" movements of Laveran's bodies are to be seen in human leucocytes, and he finds that the white corpuscles of frogs are at first intra-corpuscular, and later on free, and changing into various forms, such as crescents. Even the periodic fever is produced not by the life-changes of the corpuscles, but by the diurnal variation of the spleen and its nocturnal inactivity.

G. Thin,[3] on the other hand, argues that the whole cycle of the parasite has been watched, from the nucleated spore, not developing within a red corpuscle to begin with, but coming in contact with it, then entering it, and developing therein into separate nucleated spores. In irregular fevers the corpuscles are often localised in particular organs, and few are to be found in the peripheral circulation. This may explain the few stages seen by Lawrie in Indian cases. He is doubtless right that there probably is a swarming movement to be seen in the white corpuscles of the frog, but this is very different to the consecutive movement in fixed directions of the granules in the living malaria parasite. The sporulating stage is the important one, and it is just this which Lawrie has not seen. The latter,[4] however, later on describes a case in which a body like a red blood cell with a dark spot broke up into fourteen others, but finally the original cell wall reappeared and enclosed them all again. Manson[5] asks what was this cell wall? Has the leucocyte, such as Laveran supposes these bodies to be, a cell wall at all? This evolution of a rosette body might be only an optical phenomenon, due to an existing rosette becoming visible in the film of blood and then fading out again. Norman Moore[6] noticed in the ovoid hæmatozoon of Ashanti fever two small processes projecting from the greater curve. In other respects it much resembled the hæmamœba precox of the Roman fever. Laveran[7], from the examination of specimens from Dahomey, Tonquin, and Madagascar, believes that there is no difference in the parasites of different countries, except that under favourable conditions they may become more virulent in one place than elsewhere.

Danilewsky[8] insists that the organism of regular malarial fevers can be found in birds, and produces in them an acute form of malaria. In the irregular fevers, too, a crescent form is found similar to one met with in birds. The practical value of these various researches is shown by J. Rust.[9] A patient with phthisis suffered from remarkable pyrexia; an examination of the blood proved the presence of malaria, which was cured by quinine in large doses. The patient, who had been rapidly failing, soon gained six pounds in weight, and improved under ordinary treatment. Rottger[10] praises the action of methylene blue in malaria, which destroys the plasmodes and the fever without causing gastro-intestinal irritation. Where quinine is not tolerated, phenocoll hydrochloride, or asaprol, may be given with advantage according to Moncorvo.[11] The blackwater fever of the West Coast of Africa causes a fourth of all the deaths of the white population. It frequently comes on when the patient is recovering from an attack of another fever, and commences by a severe rigor, and the temperature curve resembles that of septicæmia. There seems to be nephritis due to the pathological secretions. Plehn's[12] description of the Ashanti parasite corresponds to that of the unpigmented parasite of Marchiafava.[13] Boison records a study of a similar hæmoglobinuric fever from Madagascar, where the corpuscles in the blood diminished by a million in twenty-four hours. Hæmoglobinæmia preceded the passage of blood pigment, and both, he considers, were caused by a splenic lesion which rendered that organ unfit to transform the hæmoglobin in the ordinary manner.

**Tetanus.**—Gibier[14] was interested by the accounts given of the malicious destruction of cattle in India by the introduction into the rectum of rags soaked in serpent venom, and he therefore tested the effect of introducing tetanus and diphtheria toxine in the same manner. To his surprise he found that large and repeated injections caused no ill effects whatever. The poison was either destroyed in the portal circulation or not absorbed Chantemesse has administered diphtheria antitoxin by the rectum with successful results, but strange to say, such injections in animals were absolutely powerless, according to Gibier. Worthington,[15] in an analysis of sixty-eight cases of tetanus, decides that the prognosis of the disease depends on the incubation period, the rapidity of onset of the spasms and their severity, and the duration of the disease. It is grave, if the incubation and the duration of the disease is short, and if the spasms are early and severe. Fifty per cent. of all the deaths occurred in two days. Antitoxin appears to have done almost nothing. On comparing these cases with a list of those treated by antitoxin, the death-rate in acute cases under antitoxin is actually higher, though more chronic cases show a reduction under that treatment.

**Leprosy.**—P. E. Todd, the superintendent of Robben Island,[16] discusses the effect of acute diseases on leprosy. An attack of erysipelas, if it causes any effect, leaves the patient worse afterwards; measles has a similar effect. Phthisis is a frequent and harmful concomitant, and it is, indeed, noticeable that the leprous tubercles on the face frequently diminish during its course. Syphilis often exists side by side with leprosy, and has no beneficial effect on the disease. In these respects his experience is completely at variance with the statements of Dr. Impey. Blaschko[17] reports the existence of the disease in the district of Memel, in Prussia. Some twenty-two recent cases were known, but whether they were imported from Russia or developed on the spot is uncertain. They are not isolated, but, according to Wasserman, various therapeutic measures are employed.

**Actinomycosis.**—A case at Glasgow was treated both by scraping and by large doses of iodine and iodide of potash without effect, and the patient gradually sank, though she had previously been a strong, healthy young woman. Knox[18] believes that the point of infection was the inside of the cheek. Another case was treated in St. Mary's Hospital, London. The patient, a single woman, aged 59, had had nothing to do with cattle, horses, or straw, and the disease commenced over the lower jaw. The bone was not implicated, nor were there any enlarged glands in the neck. The skin was widely affected, and sinuses formed from which sero-purulent matter exuded containing the rayed fungi. This implication of the skin, by the way, is not often seen in this country, though common elsewhere. Primary invasion of the skin is most rare. The patient was rapidly cured by iodide of potash, under the care of Malcolm Morris,[19] who gives a fairly full account of all that has been discovered about the disease. Most cases yield rapidly to iodide of potash, but Poncet records three others, besides Dr. Knox's mentioned above, which went from bad to worse under its use.

**Small pox.**—The present time has much of interest in relation to this disease. The Royal Commission has come to the end of its deliberations after seven years. The celebration of the centenary of vaccination has gone by, and the Gloucester epidemic has come to an end also. The majority of the Commission[20] have affirmed the value and the necessity of vaccination after the most prolonged and careful investigation, and they have attempted to render the vaccination laws as unirritating as possible. They deny the argument that smallpox depends on unsanitary conditions of life they show that a more vaccinated population, so far as this country is concerned, has shown a diminished mortality from small-pox; they urge the importance of re-vaccination, and declare that to attempt to substitute an isolation system in place of vaccination would be deplorable. Calf-lymph should, they consider, be obtainable by everyone, and to render vaccination less disliked they propose that it should be done at home at the cost of the State, under proper inspection, those persons only being excused who take the trouble to make a statutory declaration of objection to the treatment. Their suggestions are, indeed, temporary and tentative, and made with a hope to stimulate belief in the efficiency of vaccination, of which they themselves are well assured.

Instances of generalised vaccine eruption are not unknown in human beings, and, according to Copeman,[21] are frequent if colts are vaccinated. Vaccine administered by the intestinal tract to human beings will sometimes produce such an eruption. Hutchinson[22] has very rarely seen a few very large umbilicated vesicles indistinguishable from variola, but which pass away with no ill effects. However, in two cases he has seen a fatal termination after an eruption resembling variola, which followed the normal vaccinia where no infection seemed possible. Sibley brought forward a case recently[23] where general papular eruption had followed ordinary vaccinia, but had not been communicated to other children in the house. In appearance it closely resembled syphilis, though it did not affect the angles of the mouth or the anus. Copeman and Sternberg[24] suggest the possibility of perfect vaccination without any local result by the injection of sterilised or glycerised lymph. Indeed, there is some reason to think that glycerised lymph, being free from all septic and adventitious germs, should be alone used for vaccination. T. Whiteside Hime[25] gives the following hints to vaccinators: (*a*) Examine every child to see that it is in good health beforehand. Vaccination during the incubation of fevers or in the case of children suffering from or infected with hereditary syphilis may be followed by severe outbreaks according to Colcott, Fox,[26] and others; (*b*) disinfect the lancets before and after, and the child's arm beforehand, using fresh squares of tissue paper for drying it; (*c*) use blunt lancets, so as to avoid wounding the deeper layers of the skin, in which the vaccine will be inert, and do not cause any flow of blood; (*d*) endeavour to rub off the surface of the skin only with the edge of the lancet; (*e*) make at least four insertions well apart: (*f*) allow a few minutes for absorption before the child is dressed, and forbid shields; (*g*) warn the mother to keep the arm clean, but to avoid poulticing, breaking or detaching the scab; (*h*) do not be too economical in the quantity of lymph used. Sobotka[27] notices that there is a fever of remitting type from the third to the seventh day after vaccination, and a continued pyrexia from the eighth to the tenth day, which are not modified by the particular condition of the arm. Leucocytosis occurs during the remittent fever, and again, to a higher degree, during the continued one. In variola leucocytosis is slight, and disappears early. Le Dantec[28] claims to have discovered the microbe of small-pox. It is a coccus constantly present in the blood, and grows on agar or bouillon. In the latter it forms large flocculi in one or two days, which fall to the bottom and leave the bouillon clear. It renders bouillon acid and coagulates milk. Ll. Eliot[29] draws attention to the large amount of alcohol small-pox patients can take without effect, and the remarkable appetite of convalescents. In walking, patients whose prognosis is bad lift the feet very high, the eyes are drawn down, and the upper part of the white is exposed. Again, when the face assumes a leaden hue, or becomes much swollen at an early stage, a severe attack may be expected. An outbreak in the Cardiff workhouse, where 21 cases occurred out of 800 inmates, is recorded by A. Sheen.[30] Another in Guernsey, which was stamped out by rapid vaccination, is discussed by L. L. Robinson.[31] Out of 39 cases 24 were in unvaccinated people, and no one who was revaccinated beforehand took the disease. In Bombay,[32] in spite of efficient vaccination, such a large number of unprotected strangers pass through the city that compulsory vaccination for travellers staying a week is proposed, as well as general revaccination.

1 B. M. J., May 16. 2 B. M. J., May 9. 3 Lancet, May. 23. 4 Lancet, June 20. 5 Lancet, June 2. 6 Lancet, May 9. 7 B. M. J., June 6. 8 B. M. J., June 13. 9 B. M. J., June 6. 10 B. M. J., May 9. 11 Am. J. Med. Sciences, April. 12 Am. J. Med. Sciences, April. 13 B. M. J., July 11. 14 N. York Med. Jour., May 16. 15 Med. Chron., June. 16 B. M. J., June 20. 17 Med. Week, May 8. 18 Glasgow Med. Journ., May. 19 Lancet. June 6. 20 B. M. J., Aug. 22. 21 Practitioner, May. 22 Edinburgh Med. Jour., July. 23 Clinical J., July 8. 24 Med. Record, May 9. 25 B. M. J., May 23. 26 Practit., May. 27 B. M. J., May 2. 28 Practit., May. 29 N. York Med. Jour., Ap. 4. 30 B. M. J., Ap. 4. 31 B. M. J., May. 32 Indian Med, Chir. Review.

